# Distribution of T-2 toxin and HT-2 toxin during experimental feeding of yellow mealworm (*Tenebrio molitor*)

**DOI:** 10.1007/s12550-020-00411-x

**Published:** 2020-09-29

**Authors:** Nicolo Piacenza, Florian Kaltner, Ronald Maul, Manfred Gareis, Karin Schwaiger, Christoph Gottschalk

**Affiliations:** 1 Faculty of Veterinary Medicine, Ludwig-Maximilians-Universität Munich (LMU), Schoenleutnerstr. 8, 85764 Oberschleissheim, Germany; 2 National Reference Laboratory for Mycotoxins, Department Safety in the Food Chain, BfR – German Federal Institute for Risk Assessment, Max-Dohrn-Straße 8-10, 10589 Berlin, Germany; 3 Federal Research Institute of Nutrition and Food, Department of Safety and Quality of Milk and Fish Products, Max Rubner-Institut, Hermann-Weigmann-Straße 1, 24103 Kiel, Germany

**Keywords:** Yellow mealworm (*Tenebrio molitor*), Edible insects, Trichothecenes, Food safety, Mass spectrometry, Biotransformation

## Abstract

Within the European Union (EU), edible insects need to be approved as “Novel Food” according to Regulation (EU) 2015/2283 and must comply with the requirements of European food law with regard to microbiological and chemical food safety. Substrates used for feeding insects are susceptible to the growth of *Fusarium* spp. and consequently to contamination with trichothecene mycotoxins. Therefore, the current study aimed to investigate the influence of T-2 and HT-2 toxins on the larval life cycle of yellow mealworm (*Tenebrio molitor* (L.)) and to study the transfer of T-2, HT-2, T-2 triol and T-2 tetraol in the larvae. In a 4-week feeding study, *T. molitor* larvae were kept either on naturally (oat flakes moulded with *Fusarium sporotrichioides*) or artificially contaminated oat flakes, each at two levels (approximately 100 and 250 μg/kg total T-2 and HT-2). Weight gain and survival rates were monitored, and mycotoxins in the feeding substrates, larvae and residues were determined using LC-MS/MS. Larval development varied between the diets and was 44% higher for larvae fed artificially contaminated diets. However, the artificially contaminated diets had a 16% lower survival rate. No trichothecenes were detected in the surviving larvae after harvest, but T-2 and HT-2 were found both in the dead larvae and in the residues of naturally and artificially contaminated diets.

**Electronic supplementary material:**

The online version of this article (10.1007/s12550-020-00411-x) contains supplementary material, which is available to authorized users.

## Introduction

The fact that edible insects have been stated to be a potential source to reach the first three United Nations Sustainable Development Goals (no poverty, zero hunger, good health and well-being) (FAO 2009) has boosted scientific interest in not only the farming and processing of such animals but also consumer acceptance, nutritional value and safety aspects (Imathiu 2019). The economic value of insects has greatly increased, especially in Europe, and therefore, ecological and economic perspectives are part of ongoing research (Kierończyk et al. 2019; Manzano-Agugliaro et al. 2012; Yen 2015).

Many of the more than 2100 insect species referred to as edible (Jongema 2017) seem to have an adequate profile of amino acids and fatty acids as well as mineral content to serve as a diet for vertebrates, e.g. as fish feed or human nutrition (Barroso et al. 2014; Montowska et al. 2019; Yi et al. 2013). Additionally, the high protein content of insects, ranging from 52 to 76% (Zielińska et al. 2015), is seen as one of the major benefits for a future human and animal diet. Especially in Europe, concerns about the safety of insect products are leading to refusal of such products or even disgust (Bednářová et al. 2013; Hartmann et al. 2015; Yen 2009). To dissipate these concerns and develop safety schemes for farming and processing, various aspects, such as microbiology (Klunder et al. 2012), pesticides and heavy metals (Poma et al. 2017), have been investigated and addressed in European guidelines (IPIFF 2019).

Recent studies have shown that insects*—*if kept on rotten and/or mouldy substrates—may render hazardous metabolites harmless via their own metabolism or largely excrete them, especially mycotoxins, such as zearalenone or type B trichothecenes (Niermans et al. 2019; Ochoa Sanabria et al. 2019; Van Broekhoven et al. 2017). However, as shown for zearalenone, metabolism in *T. molitor* may also result in the formation of compounds of higher toxicity, such as α-zearalenol (Niermans et al. 2019). Based on these studies, it could be possible to obtain a safe product for further use for food or feed through insects reared on material no longer suitable for animal or human consumption. However, as described for aflatoxin B1 (Bosch et al. 2017) or type B trichothecenes and ochratoxins (Camenzuli et al. 2018), the various insect species appear to have different metabolization and excretion pathways for mycotoxins. However, comprehensive investigations of the metabolism and fate of trichothecenes, especially type A trichothecenes in *T. molitor*, are so far not available. In general, type A trichothecenes show several adverse effects, e.g. cytotoxicity and leukopenia in mammals (Bauer et al. 1989; Li et al. 2011), and can be found in various grain samples (EFSA 2017). Additionally, livestock farming is negatively affected by trichothecene-contaminated feed, e.g. for pigs, poultry and horses (EFSA 2011). Therefore, edible insects could be a high-value and safe food or feed alternative if trichothecenes do not accumulate, especially as the utilization of grains containing high amounts of mycotoxin of natural origin has been shown to positively affect the growth rate of *T. molitor* (Niermans et al. 2019).

The aims of the present study were to investigate the effects on the weight gain and survival of *T. molitor* larvae fed different diets containing two amounts of T-2 and HT-2 toxins. The feed was either naturally contaminated by the addition of *F. sporotrichioides*–contaminated oat flakes or artificially contaminated with T-2/HT-2 standards. After a 4-week feeding period, the occurrence of T-2, HT-2, T-2 triol and T-2 tetraol in the larvae and their residues was determined and evaluated. This study aimed to assess possible degradation pathways of type A trichothecenes in *T. molitor* larvae and to examine the ability of these species to be safe utilizers of trichothecene-contaminated diets.

## Materials and methods

### Chemical reagents and standards

The following mycotoxin calibrant solutions (Biopure™ certified reference materials, purity (HPLC) > 98.9%) were purchased from Romer Labs (Getzersdorf, Austria) and were used for all experiments and measurements. The solutions were used within the indicated expiry date: T-2 toxin (T-2, *c* = 101.2 μg/mL), HT-2 toxin (HT-2, *c* = 100.1 μg/mL), T-2 triol (Triol, *c* = 50.1 μg/mL) and T-2 tetraol (Tetraol, *c* = 50.1 μg/mL). Acetonitrile (ACN, LC-MS grade) and formic acid (p.a. grade) were purchased from Th. Geyer (Renningen, Germany) and were used for all experiments and analyses. A standard mixed solution containing all four analytical standards (*c* = 1.0 μg/mL) was prepared with ACN and stored at 6 °C in the dark. Ultrapure water was obtained using an UltraClear TM TP UV UF TM system from Evoqua Water Technologies (Barsbüttel, Germany) and was used for all experiments and analyses unless otherwise stated. Sodium sulphate (anhydrous) was purchased from Merck (Darmstadt, Germany). Ammonium formate was obtained from Fluka (Steinheim, Germany).

### Feeding substrates and diet preparation

Two different kinds of diets with two mycotoxin levels each were prepared for the feeding experiment. Diet “A” consisted of oat flakes that were artificially contaminated with T-2 and HT-2 toxins. Diet “N” was prepared by using naturally contaminated oat flakes containing a toxigenic *Fusarium* strain of mould (see below). The oat flakes used in these experiments were purchased from a local supermarket and were intended for human consumption. The levels of contamination with T-2 and HT-2 were chosen in reference to the European Commission “indicative levels for unprocessed cereals” as published by the European Commission recommendation 2013/165/EU for compound feed (EC 2013). Therefore, two diets with toxin levels of approximately 100 μg/kg (low dose) and 250 μg/kg (high dose) (sum of T-2 and HT-2 toxins) were prepared as shown in Table 1.

**Table 1:** Different diets, amounts of T-2 and HT-2 toxins and nutrient composition

Diet	Code	Biological replicates	T-2 [μg/kg]	HT-2 [μg/kg]	Dry matter [%]	Protein [%]^d^	Total energy [MJ/kg]
Natural control^a^	N(C)	2	0	0	90.1	14.3	17.8
Natural low dose^b^	N(LD)	3	88.8 ± 11.6	11.1 ± 3.0	90.6	15.5	18.1
Natural high dose^b^	N(HD)	3	262.3 ± 47.3	26.0 ± 6.6	91.1	14.3	17.9
Artificial control^a^	A(C)	2	0	0	93.3	14.5	18.3
Artificial low dose^c^	A(LD)	3	53.9 ± 3.8	51.6 ± 0.9	95.7	15.8	18.7
Artificial high dose^c^	A(HD)	3	139.8 ± 7.6	120.9 ± 2.2	95.9	14.4	18.7

^a^Uncontaminated ground oat flakes for human consumption

^b^Naturally moulded oat flakes (*F. sporotrichioides* var. *minus* Wollenweber 1930); contents adjusted by mixing and homogenization with uncontaminated ground oat flakes

^c^Ground oat flakes artificially contaminated with standards of T-2/HT-2 toxin

^d^Protein content on a dry matter basis

For preparation of the artificially contaminated diets, oat flakes were milled to flour (< 0.5 mm particle size) using a Grindomix 200 centrifugal mill (Retsch, Haan, Germany) and were contaminated with T-2 and HT-2 to obtain the diets A(LD) and A(HD). For that purpose, standards of T-2 and HT-2 were added to 200 g of oat flakes soaked in 1 L of distilled water, i.e. 10 μg of each toxin for the lower dose A(LD) and 25 μg for the higher dose A(HD). The preparations were mixed vigorously for 1 h in a 2-L Erlenmeyer flask by a magnetic stirrer and afterwards dried at 50 °C in a drying cabinet (FD-115, Binder, Tuttlingen, Germany) for 4 h and lyophilized (CTFD-10P, Berrytec, Grünwald, Germany) to a stable weight for 72 h. After lyophilization, the samples were again milled to a particle size of < 0.5 mm. For preparation of the uncontaminated control diet A(C), oat flakes were treated identically, excluding the addition of mycotoxin standards.

For preparation of the naturally contaminated diets, oat flakes (50 g) were autoclaved (121.1 °C, 15 min) with 100 mL of distilled water in an Erlenmeyer flask. Afterwards, a spore suspension of *F. sporotrichioides* var. *minus* Wollenweber 1930 (strain DSM No. 62425, obtained from the German Collection of Microorganisms and Cell Cultures – DSMZ, Braunschweig, Germany) was added. For this purpose, the strain was cultivated on malt-extract agar for 3 weeks, and spores were washed off with 2 mL of distilled water. After 2 weeks of incubation at 25 °C, the mouldy material was autoclaved again, dried and milled to flour (< 0.5 mm particle size). The naturally contaminated material contained 260 mg/kg T-2 and 18.2 mg/kg HT-2 as measured by LC-MS/MS (method described below). As a consequence of this natural co-occurrence, the same T-2/HT-2 ratio was also present in the prepared diets. Due to the high toxin amount in the moulded oat flakes, 5.0 g of the material was pre-diluted 1:40 (195 g blank milled oat flakes) and homogenized in a 500-mL polyethylene vessel by using a Reax 2 overhead shaker (Heidolph, Schwabach, Germany) for 4 h. For the diets N(LD) and N(HD), 3.2 g and 8.0 g of diluted moulded oat flakes were mixed with blank milled oat flakes to a total of 200 g and homogenized again as described above. Uncontaminated milled oat flakes were used as the control group N(C). To determine the homogeneity of the produced diets, ten samples were randomly taken from each contaminated diet and analyzed according to the LC-MS/MS protocol as given below. The results revealed relative standard deviations ranging from 10.0% for N(LD) to 19.6% for N(HD). The homogeneity was therefore considered satisfactory. The results of the controls were all < LOD for all four measured type A trichothecenes.

For quality control, the total energy and protein content in the feeding substrates were monitored (see Table 1). The total energy of the diets was determined as the heat of combustion by adiabatic bomb calorimetry (2 repetitions, IKA C2000, Rhys International Ltd, Bolton, UK) and ranged from 17.8 to 18.7 MJ/kg. For the determination of dry matter, the samples were dried at 103 °C until stable weight and weighed. The protein content was determined by the method of Dumas in a LECO FP-248 Model Nitrogen Determinator (Leco, Mönchengladbach, Germany) and ranged from 14.3 to 15.8% on a dry matter basis (Table 1).

### Selection, exposure and harvest of larvae

**Fig. 1:**
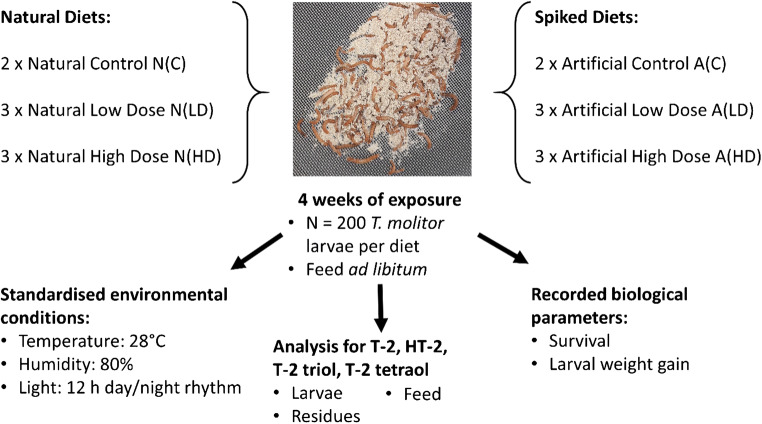
Feeding experiment design. Each diet contained n = 200 *T. molitor* larvae fed on 6 g designated feed for 4 weeks. For diet preparation and concentration, see Table 1

*T. molitor* larvae, kindly provided by the Institute for Food Technology and Biochemical Engineering of Bremerhaven University of Applied Sciences, Germany, were initially kept on wheat bran as substrate and were selected at an age of 42 days with a size of approximately 1 cm. Species identification was conducted via PCR in our laboratory (unpublished method). Before starting the feeding experiment, the larvae were starved for 48 h and divided into 16 diet groups, each containing 200 individuals with an average weight of 8.8 ± 0.3 mg per individual. The larvae were kept in 400-mL polyethylene cups for an exposure time of 4 weeks at 28 °C and 80% humidity with a 12-h day and night light rhythm. Each group was fed ad libitum with a total of 6 g of the designated diet. Experiments with the toxin-containing diets were performed in biological triplicate and blank control diets in duplicate (see Fig. 1). During the experiment, the biological parameters larval weight gain and survival rate, measured as the total amount of dead larvae for each diet, were recorded weekly. Larvae that died during the 4-week feeding experiment were separated from the living larvae and stored at − 18 °C. At the end of the exposure time, the larvae were harvested and stored—along with the residues (mixture of moults, faeces and remaining feed in the cups)—at − 18 °C before lyophilization for 72 h.

### Sample preparation of oat feed

The sample preparation procedure was based on Niermans et al. (2019) with slight modifications. Homogenous, dry oat flakes (2.0 g) were weighed in a 50-mL polystyrene tube, and 15 mL of 0.2% formic acid/ACN (50/50, v/v) was added. The samples were extracted by ultrasonication (10 min) and horizontally shaken (500 rpm, 30 min). After centrifugation for 10 min (4100×*g*, 10 °C), 1 mL of supernatant was transferred into a 2-mL reaction tube, and 250 mg of anhydrous sodium sulphate was added to separate the organic and water phases. After mixing for 30 s with a vortex laboratory shaker, the samples were again centrifuged (12800×g, 10 °C, 20 min). An aliquot of the supernatant (200 μL) was diluted with 1800 μL of water and filtered into an HPLC-glass vial using a 0.2-μm RC syringe filter (Berrytec, Grünwald, Germany).

### Sample preparation of larvae/residues

Lyophilized larval samples were ground with a mortar and pestle, and 200 mg of dry sample material was weighed in duplicate into a 2-mL reaction tube. Samples were extracted with 1.5 mL of 0.2% formic acid/ACN (50/50, v/v) in an ultrasonic bath (10 min) and horizontally shaken (500 rpm, 30 min). After centrifugation for 10 min (see above), samples were further treated as described before.

### LC-MS/MS instrumentation

A Shimadzu high-performance liquid chromatography (HPLC) apparatus including binary pumps, a degasser, an autosampler, a column oven and a control unit (LC-20AB, SIL-20AC HT, CTO-20AC, CBM-20A, Duisburg, Germany) was used for all measurements. The HPLC was coupled to an API4000 triple quadrupole mass spectrometer (MS) provided by Sciex (Darmstadt, Germany). The MS ion source parameters were set as follows: ESI + ionization voltage, 4.200 V; nebulizer gas, 50 psi; heating gas, 50 psi; curtain gas, 35 psi; temperature, 550 °C; collision gas level, 7. The MS parameters used are summarized in Online Resource 1. Data acquisition and processing were conducted with Sciex Analyst (Version 1.6.2) and MultiQuant software (Version 3.0.1).

### Measurements and quantification

For chromatographic separation of T-2, HT-2, T-2 triol and T-2 tetraol, a 50 × 2.1 mm Kinetex^TM^ 2.6 μm CoreShell EVO C18 100 Å column protected by a SecurityGuard™ ULTRA EVO C18 2.1 mm guard column (both Phenomenex, Aschaffenburg, Germany) was used. HPLC solvents were water and acetonitrile/water (95/5, v/v, B), each containing 0.1% formic acid and 5 mmol/L ammonium formate. The column oven temperature was maintained at 30 °C, and 20 μL of sample extract was injected. The binary linear gradient conditions at a flow rate of 0.4 mL/min were 0.0 min 2% B, 5.5 min 100% B, 8.5 min 100% B and additional re-equilibration of 2.2 min prior to each run. The quantification was performed via external matrix-matched calibration (linear regression). Standards were freshly prepared on each day of measurement. Aliquots of the mixed standard solution containing T-2, HT-2, T-2 triol and T-2 tetraol were pipetted into glass vials, dried under a gentle flow of nitrogen at 50 °C and reconstituted with extracts of blank oat flakes (prepared as described above) and larvae/residues obtained from the blank diets (N(C), A(C)) to compensate for matrix effects. The concentrations of the calibration standards were 0, 1.0, 2.5, 5.0, 10, 25, 50 and 100 ng/mL.

Statistical analyses of the processed data were conducted in Excel 2016 and R (Version 3.6.2, R Core Team 2019). A linear regression model was used to test the data for significance and plausibility. Figures were drawn using OriginPro software (Version 2020, OriginLab, Northampton, USA).

### Method performance and validation

Recovery rates were assessed for larvae, oat flakes and residue samples after artificial contamination of each matrix with the four analytes at two levels (75 μg/kg and 187.5 μg/kg). T-2 triol recovery in the residues was assessed by artificial contamination at the levels of 187.5 μg/kg and 300 μg/kg. Limits of detection (LOD), limits of quantification (LOQ) and linearity were calculated according to the calibration curve method of German standard norm DIN 32645 (chemical analysis—decision limit, detection limit and determination limit under repeatability conditions—terms, methods, evaluation (2008-2011)). Intraday precision (RSD_r_) was determined as the relative standard deviation of six replicates for each sample material and toxin amount. The interday precision (RSD_R_) was determined by the preparation and measurement of one sample on five consecutive days (see Table 2). Amounts smaller than the LOD were treated as “0 μg/kg”. Levels < LOQ were treated as 0.5 LOQ. All results were related to dry matter and not corrected for recovery rates.

**Table 2:** Performance parameters of the LC-MS/MS-method including recovery rates (*n* = 6 replicates) and precision

Toxin	Matrix	LOD [μg/kg]	LOQ [μg/kg]	Level of fortification [μg/kg]	Recovery [%]	RSD_r_ [%]	RSD_R_ [%]
T-2	Larvae	4.6	15.3	75	107.6	0.1	6.9
				187.5	112	1.3	7.1
	Oat flakes	3.3	11	75	101.2	4	18.5
				187.5	101.2	4	8.4
	Residue	2.1	7	75	115.4	6.1	8.6
				187.5	102.7	8.3	1.9
HT-2	Larvae	8.4	27.9	75	101.8	0.1	11
				187.5	134.3	7.2	10.2
	Oat flakes	12	39.7	75	86.1	7.7	17.3
				187.5	79.8	6.4	10.3
	Residue	20.5	68.2	75	97.7	7.5	16.9
				187.5	100	6.5	8.9
T-2 triol	Larvae	29.6	98.8	75	104.3	3.4	9.6
				187.5	105.9	4.9	7
	Oat flakes	9.5	31.5	75	75.9	11.4	25.4
				187.5	72.8	10	27.1
	Residue	69	184.4	187.5	116.1	16.3	9.5
				300	95.8	3.7	11.5
T-2 tetraol	Larvae	24.6	82.4	75	50.1	15.9	8.5
				187.5	48.5	9.5	11
	Oat flakes	30	102	75	60.1	24.1	11.1
				187.5	59.4	7.4	14.1
	Residue	25	83.7	75	50.1	22.8	10.6
				187.5	50	7.3	5.8

*LOD*, limit of detection; *LOQ*, limit of quantification; *RSDr*, intraday precision was determined as relative standard deviation by 6 replicates for each sample material and concentration; *RSDR*, interday precision was determined as relative standard deviation with one replication on 5 consecutive days

## Results

### Method performance

LODs between 2.1 μg/kg for T-2 and 69.0 μg/kg for T-2 triol (each in residues) were calculated for each single matrix (oat flakes, larvae, residues). The LOQs ranged from 7.0 μg/kg for T-2 to 184.4 μg/kg for T-2 triol (Table 2). Recovery rates (% ± RSDr) after artificial contamination of all sample materials with the four analytes ranged from 50.1 ± 22.8% for T-2 tetraol (in residue and larvae) to 116.1 ± 16.3% for T-2 triol (in residue) at the low level of fortification and from 48.5 ± 9.5% for T-2 tetraol (in larvae) to 134.3 ± 7.2% for HT-2 triol (in larvae) at the high level of fortification (see Table 2). For interday precision, RSD_R_ values from 6.9% for T-2 in larvae to 25.4% for T-2 triol in oat flakes (low level) were calculated. For the high level, RSD_R_ ranged from 1.9% for T-2 in residue to 27.1% for T-2 triol in oat flakes.

### Biological parameters

**Fig. 2:**
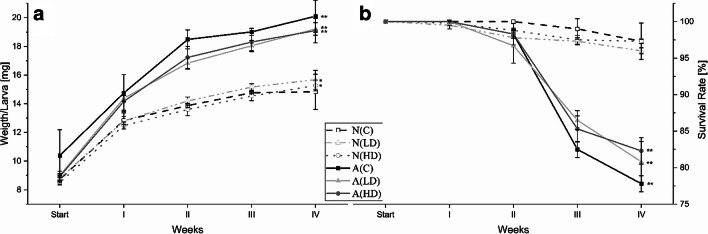
Average larval growth (A) and survival rates (B) for the different diet groups during 4 weeks of exposure to trichothecene-contaminated oat flakes (* = *P* < 0.05, ** = *P* < 0.001; linear regression model)

During the 4 weeks of exposure, the 6 feeding groups differed in their development. The larval weight gain of the artificial diets, including the control group A(C), was significantly (*P* < 0.001) higher than that of the naturally contaminated diets, e.g. larvae fed the A(HD) diet gained 44.1 ± 3% (average ± RSD) more weight than the N(C) groups (see Fig. 2 A). In total, their weight increased by 113.8 ± 1% compared with an average weight gain of 92.8 ± 21% overall diets. Moreover, larval growth differed significantly (*P* < 0.05) between diets with different toxin levels. Compared with the percentage weight gain (comparing start and end of the experiment) in the control diet (N(C)) groups, the groups fed both contaminated diets gained between 2.1 and 17.6% (N(LD)) and 7.6 and 11.0% (N(HD)) more weight. The A(LD) diet groups gained between 11.2 and 21.5% more weight than A(C) (see Table 3). The additional weight gain of the A(HD) groups ranged from 17.4 to 19.8%. Overall, larval growth was highest during the first week: larvae of all diets showed 51 ± 23% weight gain in this period, which then decreased steadily from week to week until the growth gain was 4.4 ± 36% from week 3 to week 4 (see Fig. 2 A).

**Table 3:** Larval weight gain and survival during the 4-week feeding experiment

Diet	Code/replicate No.	Weight gain [%]	Mean ± SD [%]	Weight gain RSD [%]	Survival [%]	Mean ± SD [%]
Natural control (*n* = 2)	N(C) 1	61.4	69.8 ± 11.8	16.8	94.5	97.3 ± 3.9
	N(C) 2	78.1			100	
Natural low dose (*n* = 3)	N(LD) 1	87.4	78.1 ± 8.2	10.4	94.5	96 ± 1.5
	N(LD) 2	75.2			97.5	
	N(LD) 3	71.9			96	
Natural high dose (*n* = 3)	N(HD) 1	80.7	79.3 ± 1.7	2.2	98.5	97.3 ± 1.6
	N(HD) 2	77.4			98	
	N(HD) 3	79.9			95.5	
Artificial control (*n* = 2)	A(C) 1	79	95.5 ± 23.2	24.3	76.5	77.8 ± 1.8
	A(C) 2	111.9			79	
Artificial low dose (*n* = 3)	A(LD) 1	116.9	113.3 ± 5.8	5.1	87	80.8 ± 5.3
	A(LD) 2	116.4			77.5	
	A(LD) 3	106.7			78	
Artificial high dose (*n* = 3)	A(HD) 1	113.3	113.8 ± 1.3	1.1	82.5	82.3 ± 1.8
	A(HD) 2	112.9			84	
	A(HD) 3	115.2			80.5	

After 4 weeks of exposure, larval death was observed in all blank and contaminated diets, except for one replicate of the control group N(C), in which all 200 individuals survived (Table 3). On average, the survival rate was 88.6 ± 10% (see Table 3). Compared with the control group N(C) (97.3 ± 4.0%), the survival of N(LD) (96.0%) and N(HD) (97.2%) was reduced or equal, with high biological variation, similar to that in the groups fed the artificially contaminated diets (see Fig. 2 B). However, a highly significant difference (*P* < 0.001) was observed between the natural and artificial diets (including the blank groups in both diets), resulting in an average decrease of 16.2% in the survival rate of the larvae fed the artificial diets (see Table 3).

### Occurrence of T-2 and metabolites in larvae and residues

Larvae and residues of each diet group were tested for the presence of selected type A trichothecenes. Among the samples of surviving larvae, neither T-2 nor any of its metabolites (HT-2, T-2 triol, T-2 tetraol) was detected. One pooled sample of the larvae that died during 4 weeks of exposure (dead larvae) was measured to achieve sufficient sample material for each diet group, as the cumulated dry weight of the dead larvae (72 ± 16 mg) was insufficient for assessing singular replicates.

None of the four selected trichothecenes was detected in the dead larvae of the control diet groups N(C) and A(C). HT-2, T-2 tetraol and T-2 triol were not detected in any of the dead larvae samples, but the dead larvae from the N(HD)-group contained up to 44.2 μg/kg of T-2. Lower amounts of T-2 toxin (7.7 μg/kg, > LOD - < LOQ) were found in the larvae of the diet groups N(LD), A(LD) and A(HD) (see Table 4). The control diets N(C) and A(C) were free from the investigated type A trichothecenes.

**Table 4:** Amounts of type A trichothecenes in sample material of the different diets

Sample material	Diet	Code	T-2 [μg/kg]	HT-2 [μg/kg]	T-2 triol [μg/kg]	T-2 tetraol [μg/kg]
Larvae	Natural control	N(C)	nd	nd	nd	nd
	Natural low dose	N(LD)	nd	nd	nd	nd
	Natural high dose	N(HD)	nd	nd	nd	nd
	Artificial control	A(C)	nd	nd	nd	nd
	Artificial low dose	A(LD)	nd	nd	nd	nd
	Artificial high dose	A(HD)	nd	nd	nd	nd
Dead larvae^a^	Natural control	N(C)	nd	nd	nd	nd
	Natural low dose	N(LD)	7.7^c^	nd	nd	nd
	Natural high dose	N(HD)	44.2	nd	nd	nd
	Artificial control	A(C)	nd	nd	nd	nd
	Artificial low dose	A(LD)	7.7^c^	nd	nd	nd
	Artificial high dose	A(HD)	7.7^c^	nd	nd	nd
Residue^b^	Natural control	N(C)	nd	nd	nd	nd
	Natural low dose	N(LD)	58.6^d^	nd	nd	nd
	Natural high dose	N(HD)	135.8^d^	nd	nd	nd
	Artificial control	A(C)	nd	nd	nd	nd
	Artificial low dose	A(LD)	29.7	34.1^c^	nd	nd
	Artificial high dose	A(HD)	51.1	34.1^c^	nd	nd

*nd* not detected (< LOD)

^a^*Dead larvae*, larvae that died during 4-week exposure

^b^*Residue*, mixture of oat flour and larval faeces

^c^Levels < LOQ were considered 0.5 LOQ

^d^Sample pooled to achieve sufficient material for measurement

It was not possible to investigate the larval residue separately, and neither qualitative results nor feed conversion could be derived, as the milled oat flakes had been further minced by the larvae during the feeding period, resulting in a mixture of oat flakes and larval residue at the end of the 4-week exposure time. However, compared with the fed contaminated oat diets, there was a reduction of T-2/HT-2 toxin levels in the residues (Table 4).

## Discussion

Our data revealed that the intake of type T-2 and HT-2 toxins, at total amounts of approximately 100 and 250 μg/kg, had a significant influence on the weight gain and survival of *T. molitor* larvae compared with the control groups. Additionally, the preparation method of the contaminated oat flakes affected the biological parameters, not only leading to increased body weight in the artificial diet groups (fortification of oat flake slurry with pure standard) but also to increased mortality of these larvae. With the current scientific knowledge, some of the observed effects cannot be definitely explained.

Regarding the biological parameters, Van Broekhoven et al. (2014) observed increased weight gain in *T. molitor* larvae fed T-2-contaminated diets with an almost unaffected survival rate (98%). Additionally, the results of Davis and Schieffer (1982) indicated that larval survival was not influenced by the amount of T-2, but the weight gain decreased with increasing levels of the toxin. Type B trichothecenes such as deoxynivalenol (DON) can lead to reduced larval body weight, locomotor activity and protein content (Janković-Tomanić et al. 2019). However, not only single mycotoxins can be regarded as the sole factors influencing larval biological parameters, especially since *Fusarium* spp. are able to form a variety of different toxic metabolites. For example, *F. sporotrichioides* is capable of producing at least 17 toxic metabolites (Thrane et al. 2004). The observations of larval biological parameters in the naturally contaminated diets could be attributed to the sum of existing mycotoxins, i.e., not only to the four analyzed mycotoxins but also to metabolites that have not been further investigated in the present study. Guo et al. (2014) showed that the consumption of some *Fusarium* spp. can lead to elevated mortality rates and that *T. molitor* larvae showed feed preferences resulting in avoidance of contaminated diets with potential survival threats caused by some *Fusarium* species. However, *Fusarium graminearum*–contaminated diets positively affected larval weight gain (Niermans et al. 2019), as was also observed with the *F. sporotrichioides*–contaminated diets in this feeding study. A corresponding observation from an earlier study was attributed to the fungus or other beneficial fungal secondary products (van Broekhoven et al. 2014). However, as the total energy and dietary protein can be considered equal for the different diet groups in the present feeding trial (see Table 1), the effects on larvae seemed to be due to the toxins or the different preparation methods for the diets.

T-2 is known to inhibit protein synthesis and induce apoptosis (Shifrin and Anderson 1999) in mammalian cells, e.g. in reproductive, gastrointestinal and dorsal skin tissue (Doi et al. 2006). Therefore, if T-2 is assumed to have similar effects in insect cells, the intake of trichothecenes could lead to a reduced growth rate or an increased mortality of mealworm larvae. However, biotransformation pathways for mycotoxins, in *T. molitor* or any other insect, have not yet been described, complicating the interpretation. In comparison, the T-2 degradation pathway in mammals can be divided into two phases (Bauer et al. 1989; Ueno 1984). T-2 is mainly metabolized in the intestinal epithelium and the liver and subsequently conjugated and detoxified (Bauer et al. 1989; Conrady-Lorck et al. 1988). Excretion in mammals occurs via the faeces and urine (Pfeiffer et al. 1988). As this class of insects, in particular the beetle family Tenebrionidae, has different anatomy and physiology from mammals, it is assumed that the degradation pathway of trichothecenes is also different, in particular because insects are able to detoxify natural and synthetic noxious agents in a way that is impossible for mammals (Bass et al. 2015; Ivie et al. 1983).

Although several bacteria communities are capable of metabolizing T-2 (Dohnal et al. 2008; Wachowska et al. 2017) and can be abundant in the *T. molitor* microbiome (Garofalo et al. 2019), their potential to metabolize all incidental toxins is unknown. Excretion of mycotoxins appears to occur through the larval faeces (Niermans et al. 2019; Ochoa Sanabria et al. 2019; Van Broekhoven et al. 2017). Therefore, a symbiosis of bacterial communities and larval metabolism could be possible and could lead to an unknown pathway, resulting in modification or putative degradation of T-2/HT-2 toxins in the larvae.

Another aspect that must be considered regarding larval weight gain and survival is differences in diet preparation. As the dry matter of naturally and artificially contaminated diets differed by up to 5%, it is conceivable that the larvae fed artificial diets had a higher nutrient intake due to higher dry matter and consequently higher energy density, leading to more weight gain, as was observed during the feeding experiment. Here, the larvae of A(C) gained significantly more weight than the larvae of N(C). However, as the oat flakes were prepared as a slurry in distilled water and heat treated during artificial diet preparation, it is possible that through these preparation methods, vitamins or other sensitive micronutrients were degraded or their content at least diminished. Because of the high metabolism of mealworm larvae and their repeated moulting processes, vitamin malnutrition is possible in a short time span and could lead to the increased mortality of the A(C) larvae compared with the N(C) larvae. The two diets also differed in terms of their T-2:HT-2 ratio. It cannot be excluded that these differing ratios could also have contributed to the observed differences. Consequently, diet preparation should always be considered an influencing factor in such feeding experiments.

Regarding the detection of mycotoxins, none of the four investigated trichothecenes was detectable in the living larvae. Therefore, it seems apparent that both toxins present in the diets were transformed into unknown metabolites or degraded. In the latter case, the larvae—when harvested as fit, living and authentic organisms for food production according to the general food law (EC 2002)—can be regarded as a potential degrader of T-2/HT-2 from contaminated diets. These findings are in line with the results of Van Broekhoven et al. (2017), who conducted a feeding experiment with *T. molitor* larvae grown on wheat flour naturally contaminated with mycotoxins (inter alia, the structurally similar type B trichothecene deoxynivalenol). According to their data, deoxynivalenol was not detected in the harvested larvae but was detected in larval faeces. Additionally, several other trichothecene-contaminated feeding trials have been conducted with *T. molitor* larvae (Davis and Schieffer 1982; Niermans et al. 2019; Ochoa Sanabria et al. 2019), and all pointed towards degradation of mycotoxins by the larvae. However, if unknown metabolites are formed, detoxification in the living larvae cannot be assumed as long as these compounds are not identified. To evaluate possible remaining cytotoxicity from unknown metabolites, nontargeted studies should be performed with a bioassay such as the MTT cell culture assay (Gareis 2006; Hanelt et al. 1994). Furthermore, putative chemical modifications of the toxins, e.g. adduct formation with glucuronic acid or sulphates, could be studied by incubation with the respective enzymes. The usage of radiolabelled toxins could lead to further clarification of metabolic processes as well.

Potential cross contamination could be the reason for the exclusive detection of T-2 in dead larvae, e.g. through contaminated oat particles on the larval exoskeleton or in the digestive tract. However, this appears unlikely, as no T-2/HT-2 was detected in the identically treated surviving larvae. On the other hand, it seems more likely that the biotransformation pathway of the toxins was interrupted in the dead larvae. As the sample volume of dead larvae was low and samples had to be pooled, these observations should be reassessed in further studies. A higher number of individual larvae in each biological replicate, higher toxin levels and the assessment of the influence of extrinsic factors, such as temperature and humidity, on larval metabolism would also be of high interest. As discussed before, the putative formation of unknown metabolites should also be studied to better understand the biotransformation and impact of type A trichothecenes on mealworm larvae.

When evaluating the results of the residue samples, the reduction of both T-2 and HT-2 in these samples strongly pointed towards unknown metabolic processes since the trichothecene levels of the diets can be seen as stable during the feeding trial (Omurtag 2008). If T-2 triol was excreted in low amounts during the feeding experiment, a false negative result also appears to be possible, as the validated method is quite insensitive regarding the LOD detection level in residue. Furthermore, a separate analysis of residue alone was not possible. The T-2 levels detected in dead larvae as well as T-2 and HT-2 levels in residues are—compared with the regulatory limits on toxin levels in cereals in 2013/165/EU (EC 2013)—quite low. As neither dead or perished animals nor excrements are allowed to be used or processed as food in the European Union (EC 2002), these results are interesting from a scientific point of view but have no impact on the safety of insects for food use, provided that current food legislation is respected.

In conclusion, *T. molitor* larvae were affected by the trichothecenes as well as by the diet preparation itself. Larval growth was positively influenced by the addition of trichothecene-containing *F. sporotrichioides*, but the toxin amount had no effect on larval survival within the two diet groups. The detection of T-2 in dead but not in living larvae indicated a yet unknown metabolic or biotransformation process in the larvae. Therefore, the results indicated that *T. molitor* larvae (harvested as healthy, living organisms) are potential degraders of T-2/HT-2 toxins. However, further studies on biotransformation pathways and the effects of potentially formed metabolites are still needed.

## Electronic supplementary material

ESM 1 (DOCX 15 kb)

## Abbreviations

ACN: Acetonitrile
A(C): artificial control
A(LD): Artificial low dose
A(HD): Artificial high dose
HT-2: HT-2 toxin
HPLC: High-performance liquid chromatography
LOD: Limit of detection
LOQ: Limit of quantification
MS: Mass spectrometer
N(C): Natural control
N(LD): Natural low dose
N(HD): Natural high dose
T-2: T-2 toxin
