# Supplementary material for: Distribution of T-2 toxin and HT-2 toxin during experimental feeding of yellow mealworm (Tenebrio molitor)
Source: Mycotoxin Res. 2020 Sep 29;37(1):11–21. doi: 10.1007/s12550-020-00411-x (PMC7819947; doi:10.1007/s12550-020-00411-x)
Supplement: Supplementary file 1 — (DOCX 15 kb) [file 12550_2020_411_MOESM1_ESM.docx]

### Online Resource 1 Retention times and mass spectrometric conditions for determination of investigated trichothecenes.

Article title: Distribution of T-2 toxin and HT-2 toxin during experimental feeding of yellow mealworm (Tenebrio molitor)

Journal name: Mycotoxin Research

Author names: Nicolo Piacenza^1*^, Florian Kaltner, Ronald Maul, Manfred Gareis, Karin Schwaiger, Christoph Gottschalk

^1^, Chair of Food Safety, Faculty of Veterinary Medicine, Ludwig-Maximilians-University Munich (LMU), Schoenleutnerstr. 8, 85764 Oberschleissheim, Germany

^*^Corresponding author: Nicolo Alessandro Piacenza MSc

E-mail address: Nicolo.Piacenza@ls.vetmed.uni-muenchen.de

| Compound | Abbrev. | Retention time^1^ [min] | Precursor ion [m/z] | Quantifier ion [m/z] | Qualifier ion [m/z] | DP [V] | CE [eV] (quant/qual) | CXP [V] (quant/qual) | Ion ratio^2^  (qual / quant) |  |
| --- | --- | --- | --- | --- | --- | --- | --- | --- | --- | --- |
| T-2 Toxin | T-2 | 4.9 | 484.3 | 305.2 | 245.1 | 61 | 21/19 | 18/14 | 0.79 |  |
| HT-2 Toxin | HT-2 | 4.5 | 442.2 | 263.1 | 215.1 | 51 | 19/21 | 14. Dez | 0.94 |  |
| T-2 Triol | Triol | 4.3 | 400.3 | 215.2 | 281.4 | 41 | 17/13 | Okt 26 | 0.31 |  |
| T-2 Tetraol | Tetraol | 1.4 | 316.2 | 215.1 | 233.2 | 46 | 15. Sep | 20/44 | 0.37 |  |
| *Entrance potential (EP)* = 10 V for all analytes. Abbreviations: DP: declustering potential, CE: collision energy, CXP: cell exit potential | | | | | | | |  |  | |
| ^1^ Determined using the final HPLC conditions; ^2^ Mean of three injections of a standard solution (*c* = 50 ng/mL) | | | | | | |  |  |  | |
